# Sciatic Nerve Injection Palsy in Children, Electrophysiologic Pattern and Outcome: A Case Series Study

**Published:** 2015

**Authors:** Vahideh TOOPCHIZADEH, Mohammad BARZEGAR, Afshin HABIBZADEH

**Affiliations:** 1 Physical Medicine and Rehabilitation Research Center, Tabriz University of Medical Sciences, Tabriz, Iran; 2 Pediatric Health Research Center, Tabriz University of Medical Sciences, Tabriz, Iran; 3 Medical Philosophy and History Research Center, Tabriz University of Medical Sciences, Tabriz, Iran

**Keywords:** Gluteal injection, Sciatic nerve injury, Children

## Abstract

Sciatic nerve injury is one of the frequent mononeuropathies in children that occurs due to different causes such as nerve compression, trauma and stretch during surgery. Gluteal injection is an uncommon cause of sciatic injury in developed countries. Poor techniques and frequent injections are the common cause of injection palsy. Proneal division of the sciatic nerve is more prone to injury due to anatomic and structural characteristics. The diagnosis is based on electrophysiological studies and the recovery rate is poor. In this study, in a period of 2 years between 2012 and 2013, we report seven children under 6 years old (three females and four males) with abnormal gait and foot pain following gluteal injection in pediatric electrodiagnostic center. Five children had proneal component and two with tibial component injuries. Five children were followed for one year and only one showed good recovery.

## Introduction

Sciatic nerve injury is one of the frequent mononeuropathies in children that occurs due to different causes such as nerve compression, trauma and stretch during surgery (1-3). Intramuscular gluteal injection is the most common injury mechanism of iatrogenic traumatic sciatic nerve injury, which would cause significant health problems, especially in developing countries (1-3). In children similar to adults, sciatic nerve injury usually presents with clinical features of proneal nerve involvement and electrophysiological evaluations have the main role in its diagnosis. Recovery is minimal and depends on the etiology and the severity of the nerve lesion (3-5). In this study, we report a series of sciatic nerve injury due to gluteal injection, their electrophysiological pattern and outcome.

## Case report

In a period of 2 years between 2012 and 2013, 7 children (18 months to 6 years old) with pain in lower limb, foot drop and gait problem after gluteal injection were visited in Pediatric Electrodiagnostic Center of Tabriz children hospital. The injected drugs were antibiotics, diclofenac, phenobarbital and metoclopramide (Table 1). The main complaints in all children were pain and abnormal gait after injection. In physical examination, there was weakness in ankle dorsi flexion in five children and weakness in ankle plantar flexion and knee flexion in two cases in the involved limb. Deep tendon reflex of the knee was normal in all children and the Achilles reflex of the ankle was reduced. Sensory tests were not reliable because of children’s uncooperative; however, in two patients there was pain and tenderness in the foot. In electrophysiological studies in five patients, proneal nerve was involved as having reduced or unobtainable compound muscle action potential (CMAP) of deep proneal nerve and sensory nerve action potential (SNAP) of superficial proneal nerve and slight reduction in the CMAP amplitude of tibial nerve and SNAP of sural nerve compared to uninvolved limb. In two patients with injection of phenobarbital and metoclopramide, tibial nerve was mostly involved as significant reduction in CMAP amplitude of tibial nerve and SNAP of sural nerve. Needle electromyography showed neurogenic changes in related muscles (Table 1). Five patients were followed after 12 months with electrodiagnostic tests and good regeneration was observed only in one case (Table 1). The treatment in most cases was physiotherapy and use of splint.

**Table 1:** Patients’ Characteristics with Sciatic Nerve Injection Palsy

**Patients**	**Age (y)**	**Sex**	**Drug**	**Proneal** **CMAP**	**SPN** **SNAP**	**Tibial** **CMAP**	**Sural** **SNAP**	**Neurogenic pattern in muscle**	**Regeneration** **process**
1- AJ	2	F	Diclofenac	Low Amp.	Unobtainable	Normal	Mildly low Amp.	PL, TA	Good
2- FR	3	F	Antibiotic	Unobtainable	Unobtainable	Normal	Mildly low Amp.	PL, TA	No
3- NB	1.5	M	Phenobarbital	Mildly Low Amp.	Mildly Low Amp.	Low Amp.	unobtainable	GCS	Poor
4- MA	4	F	Metoclopramide	Normal	Normal	Low Amp.	Low Amp.	GCS	A few
5- AA	3	M	Diclofenac	Low Amp	Unobtainable	Normal	Low Amp.	TA,PL	---
6- ME	7	M	Antibiotic	Unobtainable	Unobtainable	Normal	Mildly low Amp.	PL,TA	---
7- EN	2.5	M	Antibiotic	Low Amp.	Unobtainable	Normal	Mildly low Amp.	TA,PL	A few

## Discussion

The sciatic nerve is the longest and widest nerve in the body, which originates just distal to the lumbosacral plexus and is responsible for most of the function of the lower extremity. The sciatic nerve has two components, the common proneal nerve and the tibial nerve. Proneal nerve is usually more affected because of its posterolateral position and smaller amount of supporting connective tissue (5, 6). Although electrodiagnostic studies in children may be limited by poor tolerance, but they play a crucial role in establishing the diagnosis, recognizing the place, severity and type of lesion as well as recovery and prognosis of nerve injury (1, 3, 4, 7). Sciatic nerve injury due to gluteal injections is an iatrogenic injury in developing countries that could manifest as mild paresthesia to severe neurologic complications (6). In developed countries, this complication is reduced with less intramuscular injections and more attention in injection (7). The patient is usually complaining of the pain radiating to posterolateral of the limb and variable motor and sensory deficit such as parasthesia, numbness or weakness (6, 8). In this case series, we report seven children with sciatic nerve injury due to gluteal injection, including two patients with tibial nerve involvement and five patients with proneal nerve involvement. Pain and abnormal gait after injection were the chief complaint in most of the cases. In physical examination, there was weakness, pain and tenderness in sole of foot as well as reduced Achilles reflex, but normal deep tendon reflex of the knee. Srinivasan and colleagues also reported 53 sciatic nerve injuries in children in a period of 30 years and observed only one case with nerve injury due to gluteal injection (3). Unlike their study, in our series, we observed seven sciatic nerve injection injuries in two years. This case series is a report of patients in developing countries, while they reported their experience in a developed country. Understanding the anatomy of the gluteal site is important to prevent complications. In developing countries, it is possible that drugs be injected by an inexperienced person especially in private clinic, which would accompany with complications. In a recent review, Mishra et al. reported 1506 sciatic nerve injuries of which 80% were affected in childhood (9). Children are more often affected than adults because of less muscular and fat tissue relative to adults and are more prone to sciatic nerve injury (2, 3, 9). Some drugs, especially analgesics and antibiotics, have often been considered responsible for this complication; however, many other drugs were reported to cause sciatic injury (4, 9). Similarly, in our study, analgesics and antibiotics were responsible for five injuries of seven cases and metoclopramide plus phenobarbital were responsible for other two sciatic nerve injury. The reason for the injury still is not known. The thickness of subcutaneous tissue and gluteal musculature in children is a predisposing factor for the injury (6). It is presumed that allergic reaction, direct nerve fiber damage, neuronal ischemia, and constriction of scar tissue have role in the injury, besides, anatomic variations and neurotoxicity are important (1, 2). The nerve injury due to injection may cause axonal and myelin degeneration. It would also generate tissue inflammation and related inflammatory cascades which results in neuropathy and neuropathic pain (6). Recovery rate is related to the severity of the primary lesion and most of the patients had minimal recovery (3). We followed five patients for one year and recovery rate was good only in one patient. In other four cases, the recovery was minimal.

**In conclusion**, considering the high possibility of sciatic nerve injury due to gluteal injection and its poor recovery in children, it is recommended that injections be performed by an experienced person and if possible to have less intramuscular injections. In addition to proneal nerve, tibial nerve is also involved in sciatic nerve injury due to gluteal injection in children. Drugs other than antibiotics and analgesics could cause this injury.

## References

[1] Yeremeyeva E, Kline DG, Kim DH (2009). Iatrogenic sciatic nerve injuries at but¬tock and thigh levels: the Louisiana State University experience review. Neurosurgery.

[2] Bramhall RJ, Deveraj VS (2011). Traumatic sciatic nerve palsy after gluteal injection. Eur J Plastic Surg.

[3] Srinivasan J, Ryan MM, Escolar DM, Darras B, Jones HR (2011). Pediatric sciatic neuropathies: A 30-year prospective study. Neurology.

[4] Fapojuwo OA, Akinlade TS, Gbiri CA (2008). A three-year review of sciatic nerve injection palsy in the Physiotherapy Department of a Nigerian Specialist Hospital. Afr J Med Med Sci.

[5] Maqbool W, Sheikh S, Ahmed A Clinical, electrophysiological, and prognostic study of postinjection sciatic nerve injury: an avoidable cause of loss of limb in the peripheral medical service. Ann Indian Acad Neurol.

[6] Eker HE, Cok OY, Aribogan A (2010). A treatment option for post-injection sciatic neuropathy: transsacral block with methylprednisolone. Pain Physician.

[7] Yuen EC, So YT, Olney RK (1995). The electrophysiologic features of sciatic neuropathy in 100 patients. Muscle Nerve.

[8] Pandian JD, Bose S, Daniel V, Singh Y, Abraham AP (2006). Nerve injuries following intramuscular injections: a clinical and neurophysiological study from Northwest India. J Peripher Nerv Syst.

[9] Mishra P, Stringer MD (2010). Sciatic nerve injury from intramuscular injection: a persistent and global problem. Int J Clin Pract.

